# Why Did the FDA Approve These Advertisements?

**DOI:** 10.1371/journal.pmed.0030117

**Published:** 2006-02-28

**Authors:** David Oaks

**Affiliations:** **1**MindFreedom International EugeneOregonUnited States of America

I would like to thank *PLoS Medicine* for publishing Lacasse and Leo's important and methodical Essay that debunks the “chemical imbalance” advertisements for psychiatric drugs [ 1]. Why would the United States Food and Drug Administration (FDA) approve such ads? Our human rights group, MindFreedom International, has been asking that question for a long time. On behalf of MindFreedom, US Senator Ron Wyden contacted the FDA for an explanation about why they approve such advertising. In their response—which took over one year to receive—the FDA could cite no scientific literature or studies. It turns out there's a good reason that the FDA can't find any scientific evidence for the claims of a “chemical imbalance” in these ads: the scientific evidence in support of the serotonin hypothesis is very weak.

Readers who would like more information about the psychiatric industry's advertising suggesting a “chemical imbalance” in depression may be interested in the following: (1) MindFreedom's debate with Pfizer, manufacturer of Zoloft, available at http://www.mindfreedom.org/mindfreedom/pfizerlies.shtml, and (2) a historic debate with the American Psychiatric Association resulting from MindFreedom's 2003 hunger strike, available at http://www.mindfreedom.org/mindfreedom/hungerstrike.shtml (Researcher Jonathan Leo was on the MindFreedom International Scientific Panel for the hunger strike).
